# Preoperative management using Impella support for acute aortic dissection with left coronary malperfusion: a case report

**DOI:** 10.1186/s43044-024-00439-9

**Published:** 2024-01-29

**Authors:** Takahiro Shojima, Kazuyoshi Takagi, Kosuke Saku, Tomofumi Fukuda, Eiki Tayama

**Affiliations:** Division of Cardiovascular Surgery, Department of Surgery, Kurume University School of Medicine, 67 Asahimachi, Kurume, Fukuoka, 830-0011 Japan

**Keywords:** Acute aortic dissection, Coronary malperfusion, Left main trunk obstruction, Impella

## Abstract

**Background:**

Acute aortic dissection (AAD) with impaired perfusion of the left coronary artery has a poor prognosis, even after urgent radical aortic surgery, due to extensive myocardial damage. Although Impella, a microaxial-flow catheter pump, is useful in managing acute myocardial infarction, it is generally contraindicated in patients with AAD because it is an intra-aortic device and the aortic structure is compromised in these cases. Here, we introduce a novel intervention that allowed a planned aortic repair after managing circulation using Impella and venoarterial extracorporeal membrane oxygenation in a case of AAD with left main trunk malperfusion.

**Case presentation:**

A 40-year-old man presented with cardiogenic shock. Percutaneous coronary intervention was performed to address left main trunk obstruction using an intra-aortic balloon pump; however, circulatory instability persisted. The patient was transferred to our hospital after venoarterial extracorporeal membrane oxygenation. Impella CP™ was used to improve his circulatory status. However, a subsequent CT scan confirmed an AAD diagnosis. After 5 days of stable circulatory support, the patient underwent aortic root replacement and coronary artery bypass grafting.

**Conclusions:**

In patients with AAD and coronary malperfusion, adjunctive circulatory management with Impella may be a valuable therapeutic option.

## Background

Acute aortic dissection (AAD) with impaired left coronary artery perfusion usually has a poor prognosis, even after urgent radical aortic surgery, due to profound myocardial damage. Impella, a microaxial-flow catheter pump, is a useful adjunct device for managing extensive acute coronary infarction; however, its use is generally contraindicated in patients with AAD. This is because Impella is an intra-aortic device and the aortic structure is compromised in these cases. Here, we introduce a unique intervention that can allow planned aortic repair after managing circulation using Impella and venoarterial extracorporeal membrane oxygenation (VA-ECMO) in a case of AAD with left main trunk (LMT) malperfusion.

## Case presentation

**Fig. 1:**
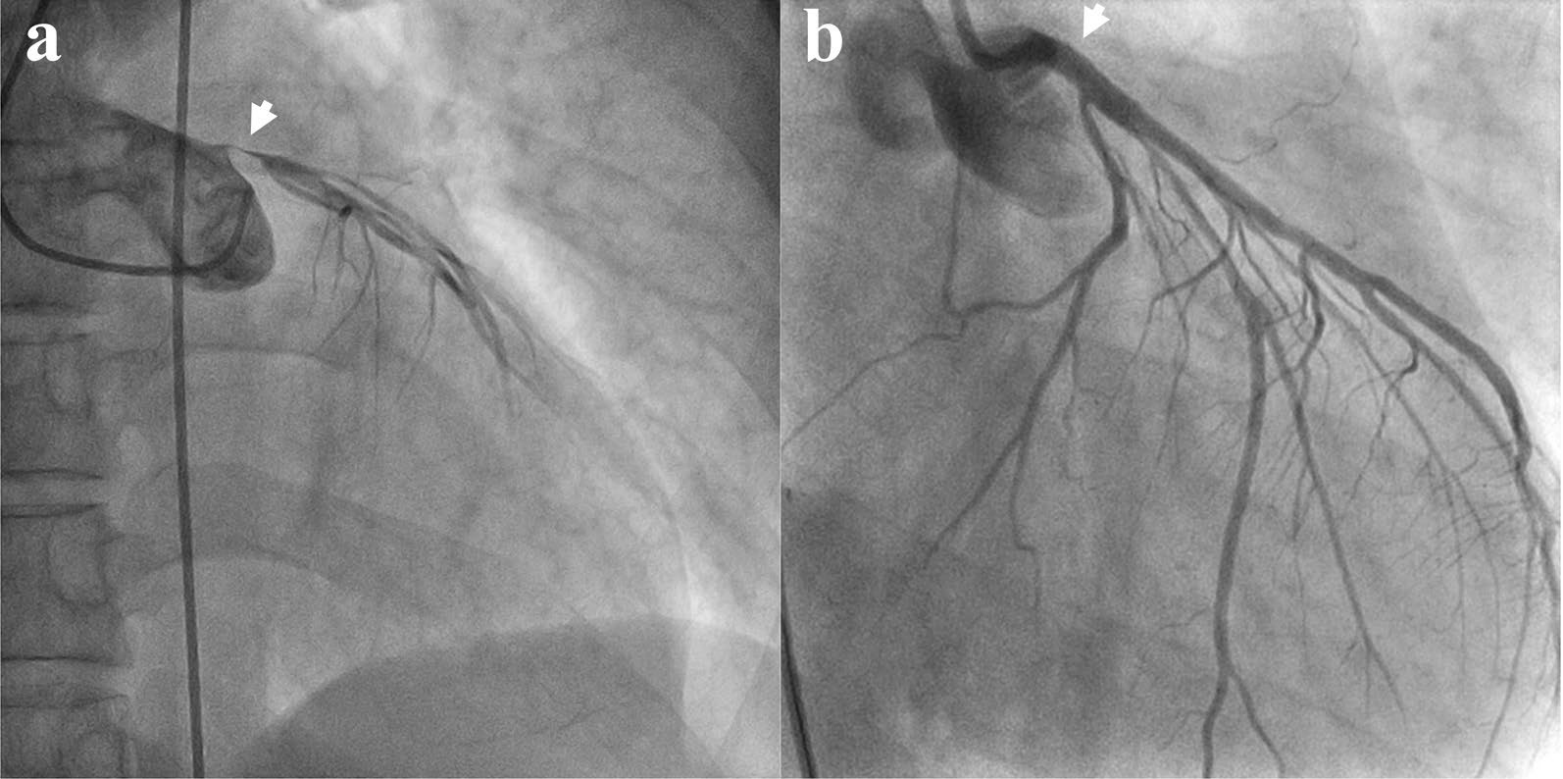
Coronary angiography. The obstructed left main trunk is shown (**a**), after being repaired using a percutaneous coronary stent (**b**)

**Fig. 2:**
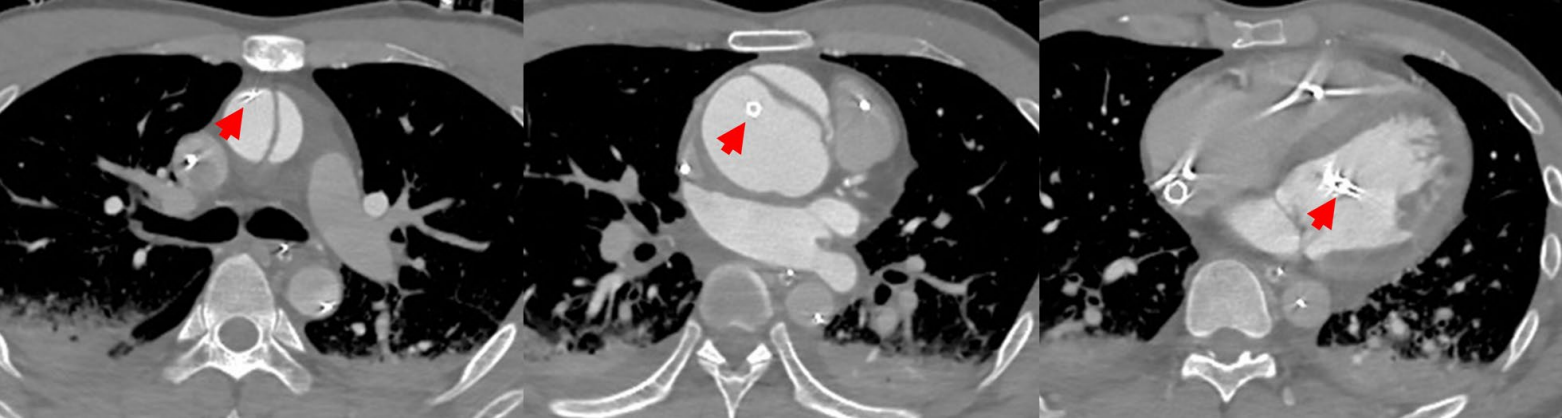
Computed tomography scan. The ascending aorta is shown after dissection and dilatation to a maximum of 52 mm. The Impella CP™ is guided through the true lumen into the left ventricle (red arrow)

**Fig. 3:**
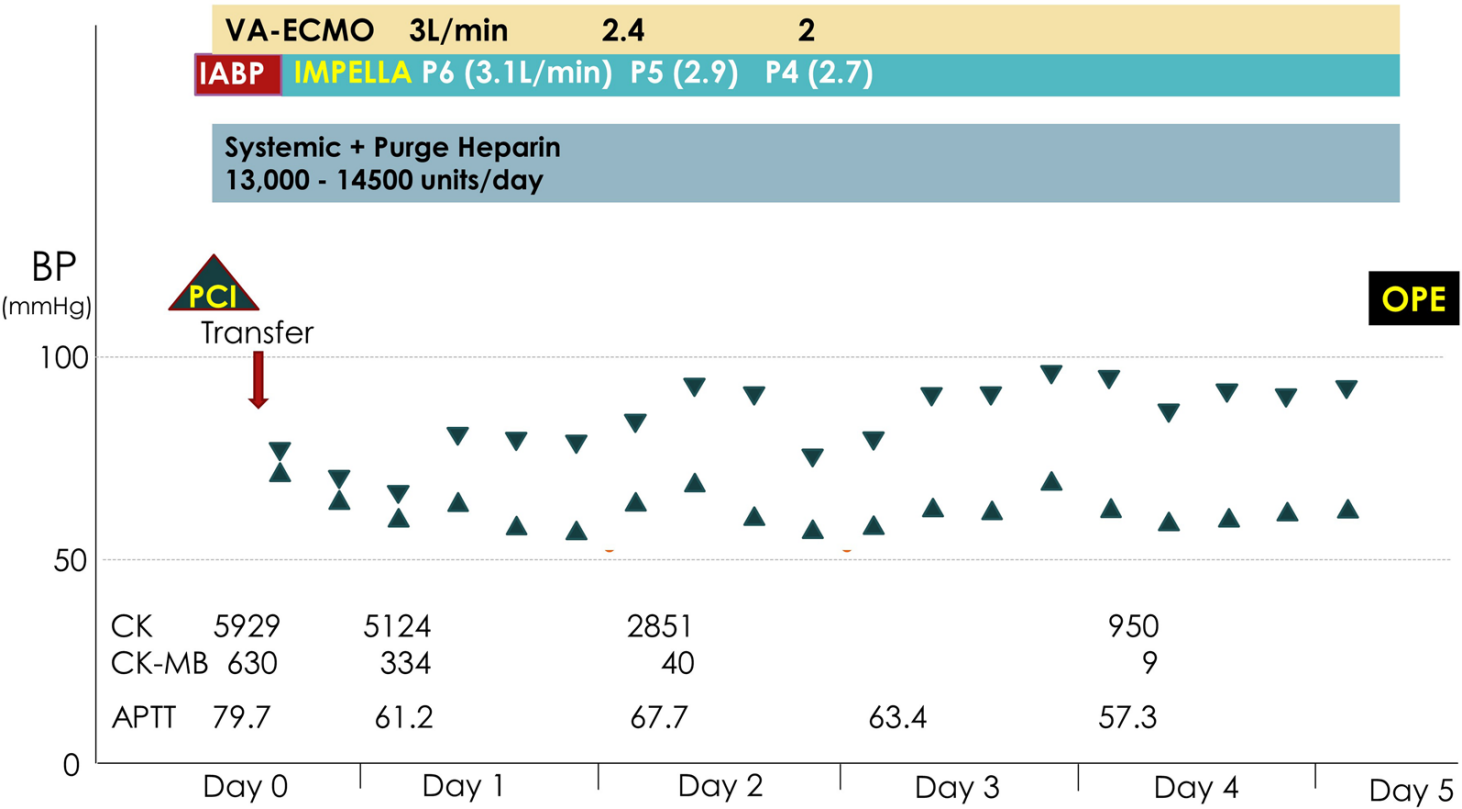
Hemodynamic time course. After Impella CP™ support, the patient’s blood pressure stabilized with a low pulse pressure. *VA-ECMO* Venoarterial extracorporeal membrane oxygenation, *IABP* Intra-aortic balloon pump, *BP* Blood pressure, *PCI* Percutaneous coronary intervention, *OPE* Operation, *CK* Creatine kinase, *CK-MB* Creatine kinase-myocardial band, *APTT* Activated partial thromboplastin time

A 40-year-old man presented to an outside medical facility with chest pain and was diagnosed with extensive anterior myocardial infarction based on the findings of ST elevation in aVR and ST depression in the inferior leads on an electrocardiogram, as well as increased creatine kinase [CK]/CK-MB levels of 3710/432 U/L, indicating acute proximal left anterior descending artery occlusion. The patient was in shock, with a blood pressure of 86/65 mmHg. Emergency coronary angiography revealed LMT obstruction. The patient then underwent percutaneous coronary intervention (Xience Skypoint 4.5 × 18 mm; Abbott Laboratories, Abbott Park, IL, USA) using an intra-aortic balloon pump (IABP) (Fig. 1). However, there was refractory shock and non-sustained ventricular tachycardia, which occurred 20 min after reperfusion. The patient was transferred to our hospital (50 km distance) after VA-ECMO insertion. Transthoracic echocardiography revealed poor left ventricular function and aortic root dilatation; however, no apparent aortic flap or pericardial effusion was observed. Consequently, we replaced the IABP with Impella CP™ (Abiomed, Danvers, MA, USA) via the left common femoral artery. Approximately 8 h afterward, contrast-enhanced computed tomography (CT) performed to delineate the morphology of the dilated aortic root revealed a dissected and dilated ascending aorta (maximum diameter, 52 mm). This led to a diagnosis of Stanford A-AAD (DeBakey type II, open type) (Fig. 2). Impella was guided through the true lumen into the left ventricle (LV). We opted to prioritize recovery from the coronary ischemic damage by maintaining the mechanical assist devices. The rate of circulatory support was 2–3 L/min on VA-ECMO and P4-6 (mean, 2.7–3.1 L/min) on Impella. Meanwhile, anticoagulant was administered at 390 U/h as a heparin-based purge solution for the Impella system and at 5,000 U/day as a systemic drug, maintaining an activated partial thromboplastin time (APTT) of 60–70 s. During support, sufficient flow was obtained with a low pulse pressure (Fig. 3). After achieving stable circulatory control for 5 days, the patient underwent aortic root replacement and coronary artery bypass grafting. The postoperative course was uneventful. After 6 months, echocardiography revealed thinning of the antero-septal wall and an ejection fraction of 45%. Moreover, the patient has been active and has no symptoms of angina pectoris or heart failure.

## Discussion

Coronary artery malperfusion in Stanford A-AAD accounts for 7–11% of cases. Although previous reports have often indicated a higher prevalence in the right coronary artery [1–3], recent studies have shown no significant difference in the occurrence of malperfusion between the left and right coronary arteries [4]. In the case of left or bilateral coronary atresia, myocardial damage can result in widespread circulatory collapse, and conventional emergency open heart surgery has poor surgical outcomes [1–3]. Performing LMT-percutaneous coronary intervention (PCI) in patients with a dissection is technically challenging; however, it is crucial to restore coronary perfusion as soon as possible [5]. In addition, a recent study reported the death of all its patients who required VA-ECMO, regardless of aortic repair [6].

Impella is a microaxial-flow pump that is inserted percutaneously through peripheral vessels to pump blood directly from the LV into the aorta. It is particularly beneficial in cases of cardiogenic shock caused by acute myocardial infarction. LV unloading during reperfusion reduces myocardial damage, decreases infarct size, and prevents progression to advanced-stage heart failure. These factors strongly support the early introduction of Impella in the treatment of acute coronary syndrome [7, 8]. In addition, when combined with VA-ECMO, Impella offsets the drawbacks of VA-ECMO, particularly the increase in LV afterload [9]. Unfortunately, it is generally contraindicated in cases of acute aortic dissection owing to its intra-aortic placement, since the aortic structure is compromised in these cases. Yoshida et al. reported their experience regarding the use of Impella in cases of a dissected aorta [10], in which the ascending aorta and aortic arches had already been repaired. To the best of our knowledge, this is the first reported case in which Impella was used prior to aortic repair in a case of AAD.

In the current case, Impella was used before the AAD diagnosis. If AAD had been identified earlier, there might have been hesitation in employing this catheter pump because of the potential risk of exacerbating the condition or causing aortic rupture. However, a previous study reported that PCI with IABP support for AAD-related LMT malperfusion was performed without adverse events [6]. In our case, the Impella was successfully inserted through the true lumen into the LV, providing circulatory support that is superior to that of the IABP and comparable to that of a normal aorta. Additionally, its lower pulsatile pressure and its ability to pump blood directly from the LV into the true aortic lumen, rather than the pseudo lumen, may have helped prevent aortic rupture.

Considering this is a single case report, we are unable to recommend early PCI and Impella support for AAD patients with LMT malperfusion. However, it is crucial to recognize that emergency open-heart surgery has a poor prognosis in critical cardiac damage cases. Thus, this clinical approach may be considered in similar cases.

## Conclusions

The use of Impella in cases of AAD demands the utmost caution. Nevertheless, in patients with AAD-related LMT malperfusion, prompt PCI and Impella support, as well as deferral of aortic surgery until restoration of cardiac function, may be beneficial. This may be especially true for patients with severe coronary insufficiency without other complications of aortic dissection, such as aortic regurgitation, rupture, tamponade, or excessive ascending aortic dilatation.

## Data Availability

Not applicable.

## Abbreviations

AAD: Acute aortic dissection
CT: Computed tomography
IABP: Intra-aortic balloon pump
LV: Left ventricle
LMT: Left main trunk
PCI: Percutaneous coronary intervention
VA-ECMO: Venoarterial extracorporeal membrane oxygenation

## References

[1] Neri E, Toscano T, Papalia U (2001). Proximal aortic dissection with coronary malperfusion: presentation, management, and outcome: presentation. J Thorac Cardiovasc Surg.

[2] Mészáros I, Mórocz J, Szlávi J (2000). Epidemiology and clinicopathology of aortic dissection. Chest.

[3] Kawahito K, Adachi H, Murata S, Yamaguchi A, Ino T (2003). Coronary malperfusion due to type A aortic dissection: mechanism and surgical management. Ann Thorac Surg.

[4] Saito Y, Hashimoto O, Nakayama T (2023). Right versus left coronary artery involvement in patients with type A acute aortic dissection. Int J Cardiol.

[5] Uchida K, Karube N, Kasama K (2018). Early reperfusion strategy improves the outcomes of surgery for type A acute aortic dissection with malperfusion. J Thorac Cardiovasc Surg.

[6] Taguchi Y, Kubo S, Ikuta A (2022). Percutaneous coronary intervention for left main coronary artery malperfusion in acute type A aortic dissection. Cardiovasc Interv Ther.

[7] Saku K, Kakino T, Arimura T (2018). Left ventricular mechanical unloading by total support of Impella in myocardial infarction reduces infarct size, preserves left ventricular function, and prevents subsequent heart failure in dogs. Circ Heart Fail.

[8] Kapur NK, Reyelt L, Swain L (2019). Mechanical left ventricular unloading to reduce infarct size during acute myocardial infarction: insight from preclinical and clinical studies. J Cardiovasc Transl Res.

[9] Patel SM, Lipinski J, Al-Kindi SG (2019). Simultaneous venoarterial extracorporeal membrane oxygenation and percutaneous left ventricular decompression therapy with Impella is associated with improved outcomes in refractory cardiogenic shock. ASAIO J.

[10] Yoshida H, Ichihara Y, Hoki R, Niinami H (2021). Impella insertion for residual aortic dissection. Clin Case Rep.

